# Molecular dynamics study of the archaeal aquaporin AqpM

**DOI:** 10.1186/1471-2164-12-S4-S8

**Published:** 2011-12-22

**Authors:** Raul Araya-Secchi, JA Garate, David S Holmes, Tomas Perez-Acle

**Affiliations:** 1 Computational Biology Laboratory, Centro de Modelamiento Matematico, Facultad de Ciencias Fisicas y Matematicas, Universidad de Chile, Santiago, Chile; 2 Facultad de Ciencias Biologicas, Universidad Andres Bello, Santiago, Chile; 3 School of Chemical and Bioprocess Engineering, University College Dublin, Dublin 4, Ireland; 4 Fundacion Ciencia para la Vida, Santiago, Chile

## Abstract

**Background:**

Aquaporins are a large family of transmembrane channel proteins that are present throughout all domains of life and are implicated in human disorders. These channels, allow the passive but selective movement of water and other small neutral solutes across cell membranes. Aquaporins have been classified into two sub-families: i) strict aquaporins that only allow the passage of water and ii) the less selective aquaglyceroporins that transport water and other neutral solutes, such as glycerol, CO_2_ or urea. Recently, the identification and characterization of a number of archaeal and bacterial aquaporins suggested the existence of a third sub-family; one that is neither a strict aquaporin nor an aquaglyceroporin. The function and phylogeny of this third family is still a matter of debate.

**Results:**

Twenty nanosecond molecular dynamics (MD) simulation of a fully hydrated tetramer of AqpM embedded in a lipid bilayer permitted predictions to be made of key biophysical parameters including: single channel osmotic permeability constant (***p_f_***), single channel diffusive permeability constant (***p_d_***), channel radius, potential water occupancy of the channel and water orientation inside the pore. These properties were compared with those of well characterized representatives of the two main aquaporin sub-families. Results show that changes in the amino acid composition of the aromatic/arginine region affect the size and polarity of the selectivity filter (SF) and could help explain the difference in water permeability between aquaporins. In addition, MD simulation results suggest that AqpM combines characteristics of strict aquaporins, such as the narrow SF and channel radius, with those of aquaglyceroporins, such as a more hydrophobic and less polar SF.

**Conclusions:**

MD simulations of AqpM extend previous evidence that this archaeal aquaporin exhibits hybrid features intermediate between the two known aquaporin sub-families, supporting the idea that it may constitute a member of a novel class of aquaporins.

## Background

Aquaporins are a large family of transmembrane channel proteins that are present throughout all domains of life and their malfunction has been implicated in several human disorders [1]. Aquaporin channels allow the passive but selective movement of water and other small neutral solutes such as glycerol, CO_2_, or urea across cell membranes [1-4].

Structurally, Aquaporins present a homotetrameric organization in which each monomer forms an individual functional pore. The canonical fold of the aquaporin monomer is characterized by a right-handed helical bundle of six transmembrane α-helices (TM1 – TM6) connected by 5 loop regions (loops A to E), in which both amino and carboxyl termini face the cytoplasmic side of the membrane. Loops B and E are formed by a half-membrane spanning helical section (HB and HE respectively) and a non-helical section that contains the highly conserved Asn-Pro-Ala (NPA) motif, considered a signature of this protein family [5-10].

The aquaporin channel posses two major constriction zones, the NPA region, located at the centre of the pore and the selectivity filter (SF) containing the aromatic/arginine region, (ar/R), located ~8 Å above the NPA region extending towards the extracellular side of the channel. The ar/R region is formed by a residue from helix TM2 (H2 position), a residue from helix TM5 (H5 position) and two residues from loop E (LE1, LE2 position respectively) [11,12]. This region plays a major role in solute selectivity and permeability [13].

Different permeability to water and other solutes and variations in the amino acid composition of the ar/R region has led to the classification of aquaporins into two main functional sub-families [14,15]. The first are strict aquaporins, that only allow the passage of water molecules, in which the ar/R region contains a Phe residue from TM2 (H2 position), a His residue from TM5 (H5), an Arg residue from the loop E (LE2) and a fourth residue from loop E (LE1) that provides a backbone carbonyl oxygen, usually a Cys, Thr or Ala. In these aquaporins, the His and Arg residues of the SF are believed to provide donor hydrogen bonds for water molecules [14,15]. Examples of strict aquaporins are: AqpZ from *Escherichia coli *[16,17], and the human Aqp0 [3], Aqp1[7] and Aqp4[18]. The second are aquaglyceroporins, less selective aquaporins that can transport water and other solutes, such as glycerol, urea and other uncharged small molecules [19,20]. In these aquaporins, the Phe residue at position H2 is replaced by Trp residue, the His residue at position H5 is replaced by Gly residue and the small residue at position LE1 is replaced by a Phe, giving rise to a wider SF and a so called “hydrophobic corner” formed by Trp (H2) and Phe (LE1) residues [3,20-23]. Similar to strict aquaporins, position LE2 is occupied by an Arg residue that is highly conserved in both aquaporin sub-families. Examples of aquaglyceroporins are: GlpF [19-21,23] from *E. coli*, and human aquaporins Aqp7, Aqp3, Aqp9 and Aqp10 [3].

The recent identification of archaeal aquaporins that exhibit a different amino acid composition of their SF, and thus cannot be readily classified in either of the two aforementioned aquaporin sub-families, has led to the suggestion that a third functional sub-family of aquaporins may exist [24,25]. It has been suggested that this family could contain special adaptations for the conduction of solutes specific for the life-style of the organisms or, alternatively, that they could be a primitive non-specialized aquaporin, an ancestor of the more specialized aquaporins [2,24,26]. The SF of these archaeal aquaporins exhibits the highly conserved Arg residue in position LE2, the Phe residue at position H2, and a Ser residue in position LE1 that provides its main chain carbonyl oxygen. However a key difference in the composition of the SF of these aquaporins lies in the presence of a medium size hydrophobic residue such as Ile, Leu or Val instead of the His residue highly conserved in strict aquaporins or the Gly residue present in aquaglyceroporins in position H5 [24,25].

To date, the only representative of this type of aquaporins that has been experimentally studied is AqpM, found in the archaeon *Methanothermobacter marburgensis *[24,25]. The osmotic water permeability constant, *P_f_*, obtained for AqpM, showed that its osmotic permeability was lower than the *P_f_* of the strict aquaporins AqpZ from *E.coli*, and slightly higher than the *P_f_* of the aquaglyceroporin from *E. coli* GlpF [24,25]. (Pf (AqpM) = 57 μms-1 [24]; Pf (AqpZ) = 330 μms-1 [27]; Pf (GlpF) = 49 μms-1 [27]). Moreover, the transient glycerol permeability measured for AqpM resulted in values considerably lower than the glycerol permeability for GlpF [24]. These previous results have led to the suggestion that AqpM could be a water transporter and also with the ability to transport other neutral solutes required by the archaeon such as CO_2_, the only carbon source available in the natural environment of M. *marburgensis*, or even H_2_S, the terminal electron acceptor in its energy production pathway [24][25].

**Figure 1:**
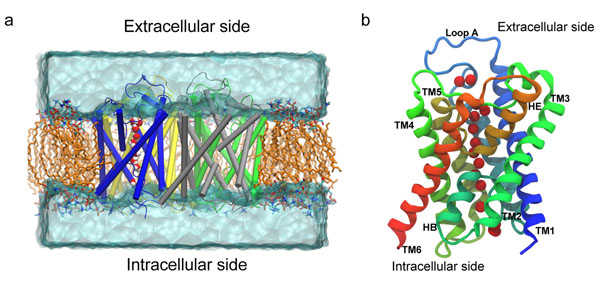
**The simulated system and the AqpM monomer.** (a) Snapshot of the Simulated System. AqpM monomers are rendered in cartoon representation. Hydrogen atoms and some lipid molecules have been omitted for clarity. Aliphatic tails of lipid molecules = orange; Lipid headgroups are colored by element (red = oxygen, blue = nitrogen, cyan = carbon). Water molecules above and below the membrane are represented as a transparent solid surface. The single file of water molecules inside one AqpM monomer is shown as vdW spheres colored by element (red = oxygen, white = hydrogen). (b) Schematic representation of the AqpM monomer viewed parallel to the membrane plane. The six transmembrane (TM1-TM6) and two half-membrane (HB and HE) spanning helices are labeled. The red vdW spheres represent the oxygen atoms of the single file of water molecules inside the AqpM monomer.

In this work we report the results of the first molecular dynamics (MD) simulation study performed on a fully hydrated model of the AqpM tetramer embedded in a lipid bilayer (as shown in Figure 1). Key biophysical parameters were estimated from the MD simulation, including: single channel osmotic permeability constant (***p_f_***), single channel diffusive permeability constant (***p_d_***), channel radius, potential water occupancy of the channel and water orientation inside the pore.

The results of this study extend the information regarding the permeability and selectivity mechanisms of AqpM and provide further insight into the water permeability of aquaporins in general and the particular differences between the sub-families of aquaporins and the implications of these differences for their function.

## Results and discussion

### Single channel osmotic water permeability constant

**Figure 2:**
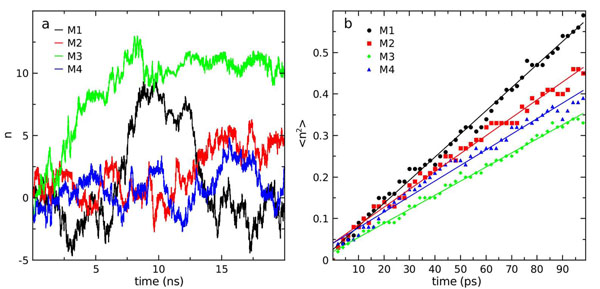
**Osmotic water permeability constant.** (a) Collective coordinate n (eq. 6) as a function of simulation time for each AqpM monomer. (b) Mean-square displacement of n <n^2^> (eq. 7) for each AqpM monomer. Each color represents an AqpM monomer (black=M1, red=M2, green=M4, blue=M4).

The single-channel osmotic water permeability constant ( *p_f_* ) is a key parameter that allows the characterization of the water transport mechanism of aquaporins [28,29]. An estimation of *p_f_* can be obtained from equilibrium MD simulations and can provide information about the water permeation mechanisms of aquaporins at the atomic level [28,30]. Here, we report the calculation and analysis of *p_f_* obtained from a 20 ns simulation of the AqpM tetramer. The calculation of *p_f_* is based on the collective diffusion model for water permeation through microscopic channels developed by Zhu et al. [30]. Initially, a trajectory for the collective coordinate *n*(*t*) was derived (Eq. 5) and the corresponding mean square displacements  were calculated (Eq. 6). Figure 2a displays the trajectory of the collective coordinates *n*(*t*) for each monomer of AqpM in the 20 ns simulation and Figure 2b shows  computed by averaging 200 100-ps time windows, each one considered as a time origin (i.e *n*(*t* = *t`*) = 0). The length of the channel is a necessary parameter for computing *n*(*t*) and was defined as the average length of the constriction region (CR) of the AqpM channel, , considered as the average distance between the atoms G195(N182/G198):O^†^ and C79(G60/A65):O, over the 20 ns simulation. The single-channel osmotic water permeability constant *p_f_* was obtained from  via *D_n_* (Eq. 6 and Eq. 7) and yields a value ranging between 5.6x10^-14^ cm^3^s^-1^ and 8.3x10^-14^ cm^3^s^-1^ among the four monomers. Averaging over the four monomers, a value of (6.4±1.4) x10^-14^ cm^3^s^-1^ is obtained. Additional details are provided in Table 1.

**Table 1:** Summary of results

	Mean ± SD	M1	M2	M3	M4
***p_d_***	1.4±1.0	2.8	0.7	1.2	0.7
***p_f_***	6.4±1.4	8.3	6.4	5.1	5.6
	7.5±0.3	7.7	7.7	7.3	7.2
***p_f_ / p_d_***	6.1±3.0	3.0	9.1	4.2	8.0
	19.9 ± 0.9	18.7	20.7	20.1	20.0

*p_f_* &*p_d_* in units of 10^-14^ cm^3^s^-1^.  is the average number of water molecules inside the channel. is the average channel length (average distance between Gly195:O and Cys79:O). Standard deviations (SD) of the mean are obtained from the variance among the four monomers (M1-M4).

Experimental measurements of the osmotic permeability of AqpM have been reported in terms of total membrane osmotic permeability constant (*P_f_* ) obtained from AqpM reconstituted into liposomes with values of 57 [24] and 60 μms^-1 ^[26]. In order to compare these values to the single-channel water osmotic permeability (*p_f_* ) values obtained in this study, an estimation of *p_f_* from *P_f_* was made using the method of Borgnia et al. [16] to estimate the *p_f_* of AqpZ [24,26]. The estimation consists in dividing *P_f_* by the number of channels per unit area (channel density). According to this, an experimentally derived estimate of the *p_f_* of AqpM is ≈ 0.7x10^-14^ cm^3^s^-1^. This suggests that the *p_f_* value obtained from the AqpM MD simulation presented here ((6.4±1.4) x10^-14^ cm^3^s^-1^) overestimates the osmotic permeability of AqpM by a factor of ~ 9. Interestingly this overestimation of *p_f_* from MD simulations has already been observed for AqpZ and other strict aquaporins and most notoriously for GlpF [28,31]. In tables 2 and 3 we present results for water permeability obtained for AqpM, Aqp1, Aqp4 and GlpF from MD simulations studies and experiments, respectively. The results in these tables show an apparent inverse relationship between experimental and MD simulation derived results. GlpF has been shown experimentally to be a slow water transporter. However in MD simulation studies it appears to be a fast water transporter, with *p_f_* values equal or greater than those obtained for prototypical and well studied strict aquaporins like AqpZ, Aqp1 or Aqp4 [28,31,32]. The cause of this discrepancy has been discussed and is attributed mainly to assumptions related to the forcefield and water model used for MD simulations and on the difficulty to determine accurately the number of active channels present when estimating *p_f_* experimentally [28,31].

**Table 2:** Comparison with other simulation results

	***p_f_***	***p_d_***	***p_f_/p_d_***			Reference
**AqpM**	**6.4±1.4**	**1.4±1.0**	**6.1±3.0**	**7.5±0.3**	**19.9±0.9**	**this work**

**AqpZ**	16±5.0	**--**	**--**	**--**	16	Hashido et al. 2005[32]
	**4.2±1.8**	**0.4±0.2**	**13.0±6.0**	**7.2±0.5**	**18.4±0.5**	**Jensen et al. 2006**[28]
	15.6±5.0	2.0±1.0	7.7±4.6	6.6±0.2	18	Hashido et al.2007 [31]

**GlpF**	16 ± 3.0	**--**	**--**	**--**	**--**	Hashido et al. 2005[32]
	14.0± 0.4	**--**	**--**	**--**	**--**	Zhu et al. 2002 [33]
	**13.1±3.4**	**3.2±0.8**	**2.9±0.8**	**8.6±0.3**	**19.6±0.1**	**Jensen et al. 2006 **[28]
	15.8±2.8	3.5±1.4	4.6±2.0	7.8±0.2	18	Hashido et al.2007 [31]

**b-Aqp1**	10.1±4.0	1.4±1.0	7.1±5.9	6.9±0.4	18	Hashido et al.2007 [31]
	10±4.0	--	--	--	16	Hashido et al. 2005[32]
	7.1±0.9	--	--	--	15	Zhu et al. 2004 [29]

**r-Aqp4**	7.0±2.8	1.0±0.6	6.9±5.0	6.9±0.5	18	Hashido et al.2007 [31]
**h-Aqp4**	2.9±0.5	0.72±0.2	--	5.8±0.7	18	Garate, J.A. et al [34]

*p_f_* and *p_d_* in units of 10^-14^cm^3^s^-1^.  average number of water molecules inside the channel (CR).  average length of the CR in Å.

**Table 3:** Experimental results

	***p_f_***	***P_f_***	Method	Ref.
**AqpM**	0.6^*^	5.7x10^-3^	Liposomes	Kozono et al. 2003 [24]
	0.7^*^	6.0x10^-3^	Liposomes	Lee et al. 2005 [26]

**AqpZ**	2.0		Planar lipid bilayer	Pohl et al. 2001[62]
	≥10.0		Liposomes	Borgnia et al. 1999 [16]
		3.3x10^-2^	Liposomes	Borgnia et al. 2001 [27]

**GlpF**	0.7		Planar lipid bilayer	Pohl et al 2005 [63]
		4.9x10^-3^	Liposomes	Borgnia et al. 2001 [27]

**h-Aqp1**	4.6		Liposomes	Zeidel et al. 1994[64]
**h-Aqp1**	5.43	0.472	Liposomes	Walz et al. 1994 [65]
**r-Aqp1**	6	19x10^-3^	*Xenopus* Oocytes	Yang et al. 1997[66]
**h-Aqp1**	11.7	--	Liposomes	Zeidel et al. 1992 [67]
**h-Aqp1**	0.96		Liposomes	Tanimura et al. 2009[68]

**r-Aqp4**	24	10x10^-3^	*Xenopus* Oocytes	Yang et al. 1997 [66]
	3.5 - 9		*Xenopus* Oocytes	Jung et al. 1994 [18]
	1.5		Liposomes	Tanimura et al. 2009[68]

^*^ Obtained from P_f_ using the method reported in [16,27].

*p_f_* in 10-14 cm^3^s^-1^. Single channel water permeability constant.

*P_f_* in cms^-1^. Osmotic water permeability constant.

An examination of the published MD simulation results obtained for representative aquaporins from both sub-families, including AqpZ [28,31,32] and GlpF [28,31-33] from *E.coli*, and mammalian Aqp1 [29,31,32] and Aqp4 [31,34], revealed significant discrepancies on the values obtained for the osmotic and diffusive water permeability and the *p_f_*/*p_d_* ratio for the same aquaporin between different studies (see Table 2). These discrepancies may arise from the use of different force-fields, water models, simulation protocols, simulation times, and insufficient sampling due to the limited time scale of all-atom MD simulations [31]. This issue together with the fact that only one representative from the aquaglyceroporins sub-family, GlpF from *E. coli*, has been subjected to MD-simulation studies, whereas four representatives from the strict aquaporins have been widely studied (Aqp0, Aqp1, Aqp4, AqpZ) exacerbates the problem of aquaporin sub-family comparisons in terms of water permeability and thus difficult the interpretation of the data obtained for newly described aquaporins such as AqpM in terms of water permeability. In an attempt to overcome these difficulties and to establish a clear point of reference, the present study of AqpM was performed using simulation protocols and analyses methods similar to those previously used in the study of AqpZ and GlpF [28,35], including: i) the use of the same force-field (CHARMM22 for protein and CHARMM27 for lipids), and water model (TIP3P), ii) similar simulation protocol (NPT ensemble, Periodic Boundary Condition, no constraints applied in the production run), iii) similar simulation time (20ns) and temperature (310K) and iv) the same MD engine (NAMD) (see methods). The comparison results in the following trend: *p_f_* (AqpZ) <*p_f_*(AqpM) <*p_f_*(GlpF) (see table 2) indicating that the single channel osmotic water permeability of AqpM presents a value of *p_f_*that is intermediate between those obtained for AqpZ and GlpF. As a whole, this evidence suggests that AqpM exhibits water osmotic permeability that is intermediate between strict aquaporins and aquaglyceroporins and thus could belong to a third sub-family of aquaporins.

### Single-channel diffusive water permeability constant

**Figure 3:**
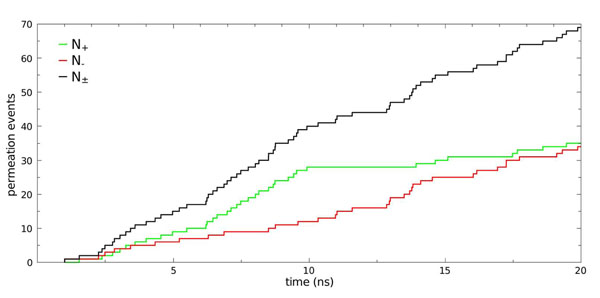
**Permeation events.** Number of permeation events along +z (**N_+_**) and -z (**N_-_**) and their sum (**N_±_**) as a function of simulation time for the simulated AqpM tetramer. Permeation events were counted from 10,000 snapshots separated by 2ps taken from the 20 ns simulation.

The single channel diffusive water permeability constant *p_d_* is another key feature that describes the water permeation mechanism of aquaporins, and is related to the number of water molecules that traverse the channel per unit time (i.e the number of permeation events)[28,36]. In the 20 ns simulation reported here, a total of 69 permeation events were observed (N_±_), with N_+_ = 35 and N_-_= 34. The accumulated number of permeation events grows linearly with time and the number of permeation events occurring in either direction is as expected, since in an equilibrium MD simulation no net water flow should be present (Fig. 3). Then the unidirectional rate constant (*k_0_*) was determined by using: *k_0_*=N_±_/2nmt_sim_, where N_±_ is the number of accumulated bidirectional permeation events, t_sim_ is the simulation time discarding the first ns (tsim = 20-1 = 19 ns) and **nm** represents the number of monomers (nm=4). Using *k_0_* (*k_0_* = 0.5ns^-1^) and Eq. 3, a value of *p_d_*= 1.4 x10^-14^ cm^3^s^-1^ was obtained for the AqpM tetramer. Additional details and *p_d_* estimates for each monomer are provided in Table 1.

As noted for *p_f_* , different MD simulation studies of the same aquaporin show significant discrepancies between the values obtained for *p_d_* (see table 2). However, despite these discrepancies a trend is apparent, in which GlpF appears with the highest *p_d_* values with respect to all other aquaporins including AqpM. The observation that the higher transport rate appears to be in an opposite trend with respect to experimental results can be explained by the wider pore of GlpF that could allow more water-water interchanges and thus more permeation events per unit time, increasing the value of *p_d_* (see below). As mentioned above for *p_f_*, this fact can be mainly attributed to the nature of MD simulations, and specifically to the water model used, the van der Waals parameters of the forcefield, and non-physical breathing movements of the channel. Following the argument used for the comparison of *p_f_* values, we obtained for *p_d_* the same trend observed for *p_f_*(*p_d_*(AqpZ) <*p_d_*(AqpM) <*p_d_*(GlpF). This result indicates that the single channel diffusive water permeability of AqpM presents values that are intermediate between those obtained for AqpZ and GlpF, using equivalent simulation methods [28]. This observation, together with the results observed for *p_f_*, provides the evidence to support the notion that AqpM could be a representative of a third aquaporin sub-family that exhibits properties that are intermediate between those of strict aquaporins and aquaglyceroporins.

### *p_f_* / *p_d_* ratio

According to the continuous time random walk (CTRW) model developed for single-file water transport [37], the *p_f_ / p_d_* ratio is related to the number of steps that a water molecule must perform in order to traverse the channel and follows the relation: [37,38] were  is the average number of water molecules inside the channel. Thus the *p_f_ /p_d_* ratio can be used as a measure of the “single-fileness” of water movement inside the channel, and for a channel in which the water molecules are perfectly aligned in single file [28,37,38]. Values of  indicates that the single-fileness of the water molecules inside the channel is interrupted by occasional water-water interchanges that increase *p_d_*, i.e., more water molecules traverse the channel per unit time [28,37,38]. Water-water interchanges do not affect *p_f_* because the latter is related to the displacement of individual water molecules inside the channel and is not influenced by the occurrence of water-water interchanges. From the 20 ns simulation of the AqpM tetramer, we obtained 3.0 <*p_f_ / p_d_* < 9.1 with an average value over the four monomers of *p_f_* / *p_d_* ≈ 6.1 and an average channel occupancy equal to  (see below). These results suggest that water molecules move in single file in the AqpM channel with occasional water-water interchange events.

For AqpZ , Aqp1 and Aqp4, values of  have been reported, whereas for the aquaglyceroporin GlpF [28,31]. This indicates that strict-aquaporins, with their narrower pore posses a more idealized single-file water transport mechanism. Interestingly, this relation holds despite the aforementioned discrepancies between the values for *p_d_* and *p_f_* observed between different MD-simulation studies. Our results shows that, in terms of the *p_f_ / p_d_* ratio, AqpM presents a similar level of single-fileness with respect to strict aquaporins (see table 2) but with an average value of  suggesting that AqpM allows more water-water interchanges than strict aquaporins. This could be explained by the slightly wider ar/R region of AqpM due to the replacement of the His residue in position H5 conserved in strict aquaporins by the smaller and apolar Ile residue.

### Channel occupancy

**Figure 4:**
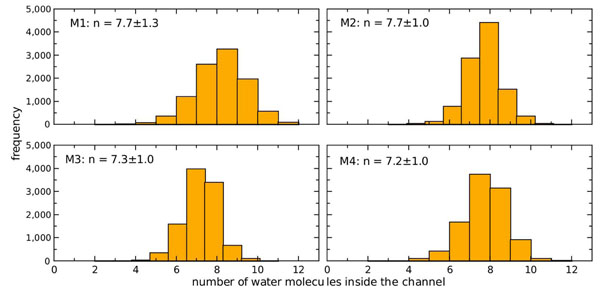
**AqpM channel water occupancy.** Water occupancy histograms for the four AqpM monomers (M1-M4) are shown. The number of water molecules inside the constriction region of each AqpM monomer was determined for 10,000 snapshots separated by 2 ps taken from the 20 ns simulation. The average occupancy *n* of each monomer is displayed on the upper-left corner of each panel. The histograms show that the four monomers have an average of 7±1 water molecules inside the constriction region.

Water occupancy histograms from the 20 ns simulation of the AqpM tetramer are shown for each monomer in Figure 4, and the averages are listed in Table.1. Histograms were generated by counting the number of water molecules inside the constriction region (CR) of each AqpM monomer for 10000 snapshots separated by 2 ps taken from the 20 ns MD simulation. The four monomers show a similar behaviour with their occupancy fluctuating closely around the tetramer average water occupancy  (Fig.4). The value of water occupancy reported here for AqpM is in agreement with results obtained from similar MD simulations of other aquaporins (see Table 2) [28,31,35] and for water transport in carbon nanotubes of similar length [37,38] in which a value of  was found.

### Channel radius

**Figure 5:**
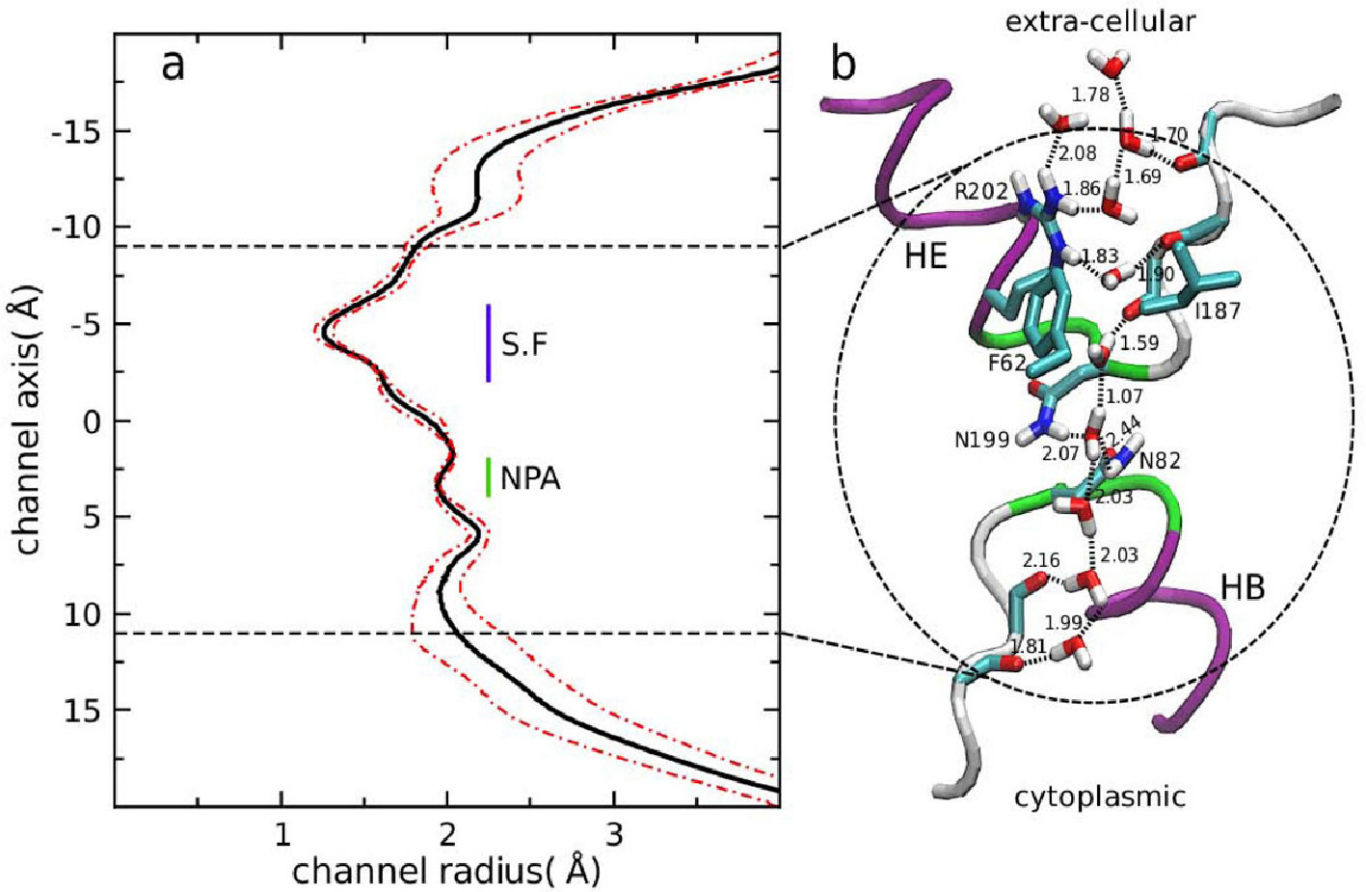
**The constriction region of the simulated AqpM channel. (*a*)** Channel radius calculated with HOLE [39]. The continuous black line represents the radius average over 10000 snapshots of each monomer (40000 snapshots) separated by 2 ps taken from the 20 ns simulation. The red dashed lines represent the channel fluctuations (standard deviation). The horizontal black dashed lines represent the boundaries of the constriction region (-9 Å < z < 11 Å) (zone surrounded by a dashed circle in *b*). The horizontal blue line indicates the selectivity filter (SF) located in the extra-cellular side (-6 Å < z < -2 Å) that comprises the narrowest part of the channel. The green vertical line indicates the position of the NPA region (2 Å < z < 4 Å) where a minor narrowing of the channel occurs. **(*b*)** Snapshot of the AqpM constriction region. The half-helices of the re-entrant loops HE and HB are shown as purple ribbons. The non-helical parts of the loops are shown as white ribbons and the main-chain carbonyl groups that form the hydrogen-bond ladder of the AqpM channel are colored by element (cyan = carbon, red = oxygen, blue = nitrogen). Potential hydrogen bonds between carbonyl groups and water molecules and between the water molecules are shown as black dashed lines. Numbers below the lines represent the average distance between the atoms involved in the hydrogen bond (in Å). The residues comprising the selectivity filter (Phe 62, Ile 187, Arg 202) are colored by element (cyan = carbon, red = oxygen, blue = nitrogen) and non-polar hydrogen atoms were removed for clarity. The two NPA motifs are shown as green ribbons and the sidechains of Asn 82, and Asn 199 are colored by element as described above and non-polar hydrogen atoms were removed for clarity. The region surrounded by a black dashed circle corresponds to the constriction region of the AqpM channel.

The channel radius of AqpM was computed using the HOLE program [39]. A total of 10000 configurations separated by 2 ps of each AqpM monomer taken from the MD simulation were considered, yielding an average channel radius of 1.9±0.4 Å for the AqpM constriction region (CR) (-9 Å < z <12 Å). A plot of the radius profile obtained from the 20 ns simulation accompanied by a snapshot of the AqpM constriction region showing the conformation and position of relevant residues are presented in Figure 5a and Figure 5.b, respectively. The channel radius value reported here is in agreement with the channel radius measured from the x-ray structure of AqpM [26]. Comparison of the channel radius profile obtained for AqpM with channel radius measurements with similar methods for other MD-simulated aquaporins, shows that the AqpM channel exhibits an average radius similar to AqpZ ≈ 1.9 Ǻ but is narrower than the average channel radius (≈ 2.5 Ǻ) of GlpF [28,31,32]. As reported for other aquaporins [28,32,35], the minimum radius of the AqpM channel is found at the extracellular side in the selectivity filter (SF) or ar/R region composed of residues R202(R189/R206)^†^, F62(F43/W48) and I187(H174/G191) with an average value of 1.4 Å, close to the radius of a single water molecule. This narrowing of the pore at the SF, together with the small fluctuations (RMS) during the MD simulation found on this region (Fig. 5a), suggest that only water and small neutral solutes with similar radius such as CO_2_ or H_2_S could potentially pass through the selectivity filter of AqpM [26]. However, it has been shown recently that AqpM is not an H_2_S transporter [25], thus more experimental and theoretical studies of AqpM are required to determine whether this aquaporin can transport other solutes through its channel.

### Water orientation

**Figure 6:**
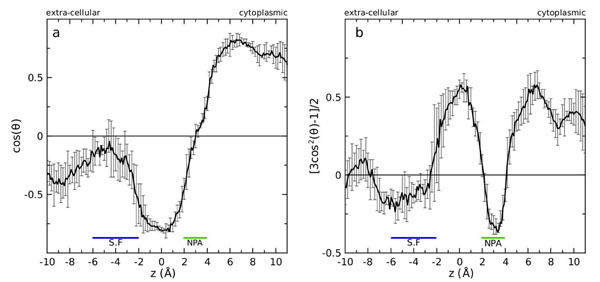
**Water orientation inside the constriction region of the AqpM channel.** (a) Order parameter . (b) Order parameter . *θ* is the angle between the unit vector, approximately aligned along the channel axis/membrane normal, **n**_z_, and the water dipole vector. Both parameters were measured for each AqpM monomer from 10000 snapshots separated by 2 ps taken from the 20 ns simulation. The black continuous line in each panel represents the average over the 10,000 snapshots and over the four monomers. The error bars represent the standard deviation from the average.

To measure the degree of ordering and orientation of water molecules inside the constriction region of AqpM, two order parameters  and , were measured. In both parameters θ is the angle between the unit vector approximately aligned along the channel axis/membrane normal, , and the water dipole vector. Both parameters *P_1_*(*z*) and *P_2_*(*z*) serve as indications of the average alignment and orientation of the water dipole relative to the channel axis and thus permit the prediction of the water orientation inside aquaporin channels[31-35]. The order parameter *P_1_*(*z*) ranges from -1 to 1, and shows the average orientation of the water dipoles with respect to the channel axis . Negative values indicate that the water dipoles are aligned parallel to the channel axis and pointing towards the intracellular side of the channel. Positive values indicate that the water dipoles are aligned parallel to the channel axis and pointing towards the extracellular side of the channel. Values of *P_1_*(*z*) near or equal to 0 indicate a perpendicular orientation of the water dipoles with respect to the channel axis. On the other hand *P_2_*(*z*), ranges from -0.5 to 1 and positive values indicates an average preferential alignment of the water dipoles parallel to the channel axis , and negative values are an indication of preferential alignment of the water dipole perpendicular to . As can be inferred from an inspection of Figure 6, water molecules appear to be highly ordered within the constriction region (CR) of the AqpM pore and a bi-polar orientation of the water dipoles with a dipole inversion at the NPA region can be observed, where both a minimum of *P_2_*(*z*) (Fig. 6b) and values near 0 for *P_1_*(*z*) (Fig. 6a) are found, indicating that water dipoles are oriented perpendicularly to the channel axis in this region. Water molecules are aligned parallel to the channel axis on both sides of the NPA motifs with their dipoles pointing towards the NPA region. It has been suggested that the bi-polar orientation of water inside the pore of aquaporins is related to the blocking of proton passage through the channel and that could involve a hydrogen donor/hydrogen acceptor pattern that probably induce the dipole inversion around the NPA motifs [40]. The nature of this bi-polar water orientation has been a matter of debate [41-44] and the current hypothesis is that the bi-polar water orientation itself is not part of the proton blocking mechanism but rather is a signature, an indirect effect of the electrostatic field generated by the two macro-dipoles of the helical part of the re-entrant loops B and E. These macro-dipoles produce a concentration of positive charge in the NPA region of the channel that generates an energetic barrier for protons blocking their passage through the pore [44].

A bi-polar water orientation is clearly observed inside the AqpM constriction region in the course of our MD simulation (Fig.5b and Fig.6) and the hydrogen donor/hydrogen acceptor pattern appears to be formed by: i) the main chain carbonyl groups exposed to the channel lumen at both sides of the NPA region provided by residues G195(N182/G199), S196(T183/F200), S197(S184/A201) on the extra-cellular side, and residues H80(H61/H66), C79(G60/A65) on the intra-cellular side, that could act as hydrogen bond acceptors forming an hydrogen-bond ladder within the pore and ii) the Asn residues at the NPA region N199(N186/N203) and N82(N63/N68) that act as hydrogen bond donors. An increase in the values of *P_1_*(*z*) and negative values of *P_2_*(*z*) are observed in the SF of AqpM, indicating that water molecules in this region orient their dipoles perpendicular to the channel axis. This could reflect the interaction of water molecules with the polar hydrogen atoms Arg202(R189/R206):H_η_ and Arg202(R189/R206):H_ε_ that causes a change in the dipole orientation of water molecules in this region. This interaction appears to be enhanced due to the narrowing of the channel in the SF of AqpM. Thus the unique amino acid composition of the SF of AqpM appears to influence the water orientation in this zone of the channel. Calculation of the same order parameters for other MD-simulated aquaporins indicate that, in the case of strict aquaporins like AqpZ and Aqp1, water molecules assume a more perpendicular orientation of their dipoles with respect to the channel axis around the SF due to the narrowing of the pore and the strong interaction between water molecules and the Arg (LE2 position) and His (H5 position) residues that comprise the SF of strict aquaporins [28,31,32]. On the other hand, the wider and hydrophobic SF of the aquaglyceroporin GlpF allows more water-water interactions and thus favours a water dipole orientation parallel to the channel axis [31,32,35].

### Structure / sequence comparisons

**Figure 7:**
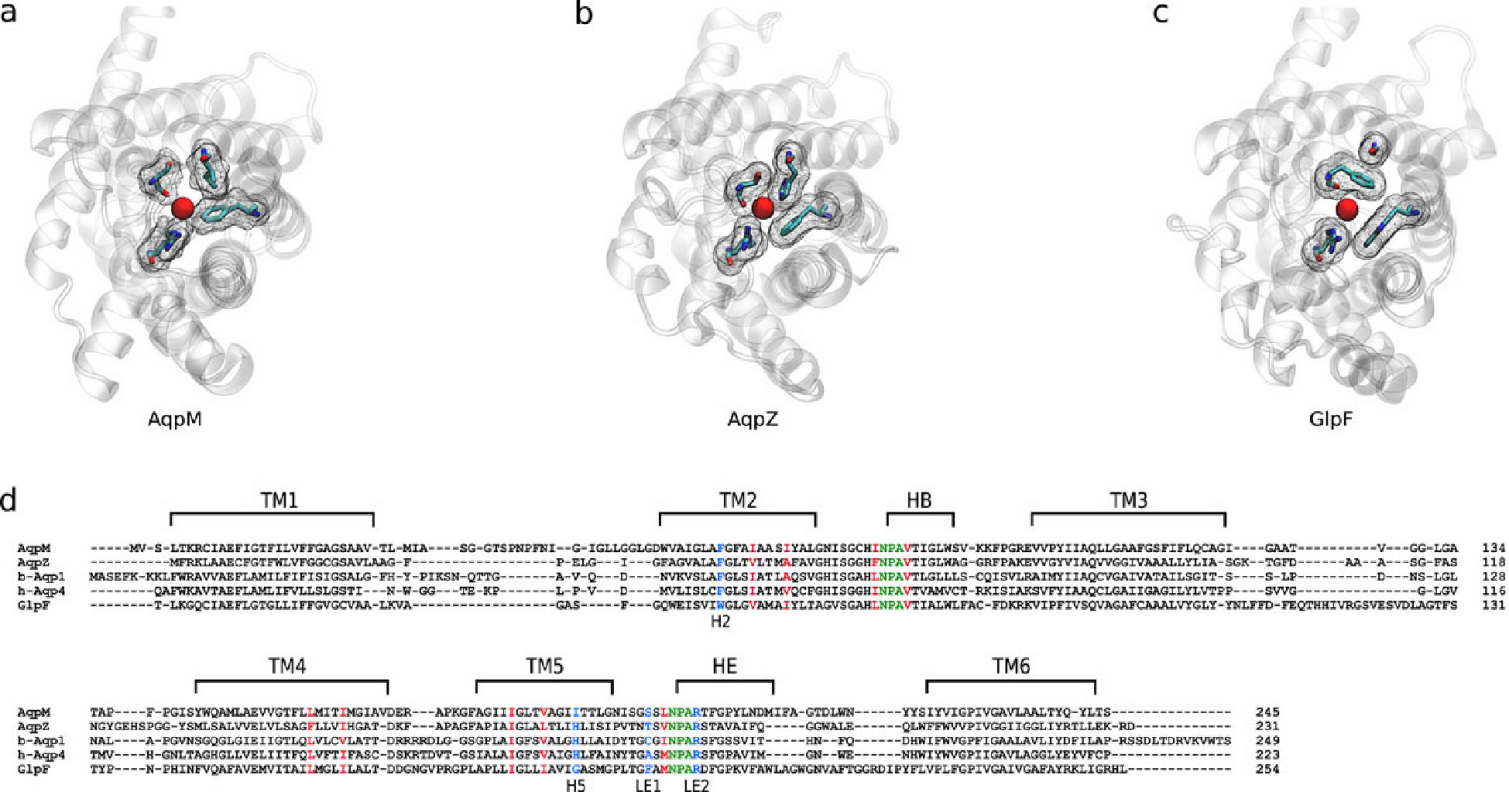
**Structure / sequence comparisons.** (a) Extracellular view of the selectivity filter of an average conformation of the simulated AqpM, (b) the crystal structure of AqpZ [PDB-ID: 1RC2(A chain)] and (c) GlpF [PDB-ID: 1LDI]. Structures were aligned with STAMP [47] (as shown in (d) below). The protein structures are represented as transparent ribbons. Residues comprising the selectivity filter of each aquaporin are colored by element (cyan = carbon, red = oxygen, blue = nitrogen) and surrounded by a mesh surface. The red sphere in the center of each pore represents the oxygen atom of a water molecule in vdW representation. (d) Sequence alignment produced using STAMP [45] of an average structure of AqpM, the crystal structure of AqpZ [PDB-ID: 1RC2(A chain)], b-Aqp1 [PDB-ID: 1J4N], h-Aqp4 [PDB-ID: 3GD8] and GlpF [PDB-ID: 1LDI]. Horizontal black lines labeled TM1 to TM6 indicate the consensus transmembrane helical regions of the five aquaporins. HB and HE correspond to the half-helices from the re-entrant loops B and E, respectively. NPA motifs are highlighted in green, residues belonging to the selectivity filter (positions H2, H5, LE1 and LE2) are highlighted in blue and hydrophobic residues that line the pore below the SF are highlighted in red.

The average structure of AqpM was compared with the crystal structures of AqpZ [PDB:1RC2 (A chain)] [17], b-Aqp1 [PDB:1J4N][45] , h-Aqp4 [PDB:3GD8][46] and GlpF [PDB:1LDI] [40] using STAMP [47,48]. Fig.7a-c compares the view from the extracellular side of AqpM (Fig.7a) with that of AqpZ (Fig.7b) and GlpF (Fig.7C); the latter were selected as prototypical representatives of the strict aquaporin and aquaglyceroporin sub-families respectively.

As can be observed in Figure 7(a-d), there are significant differences between the compared aquaporins, regarding the residues that comprise their respective SFs and their spatial arrangement (residues highlighted in blue in Fig. 7d). Of particular interest, is the residue that occupies position H5 (TM5). In AqpZ, b-Aqp1 and h-Aqp4 this position is occupied by a His residue conserved among strict aquaporins [8](Fig.7d). In GlpF and other aquaglyceroporins position H5 is occupied by a Gly, a feature that allows the formation of the so-called “hydrophobic corner” by Trp48 (replaced by a Phe residue in AqpM and in strict aquaporins) in position H2 and Phe200 (a Ser residue in AqpM, a Tyr residue in AqpZ, a Cys residue in b-Aqp1 and an ala residue in h-Aqp4) in position LE1 that allows the accommodation of a glycerol molecule [19,20,49]. In AqpM, position H5 is occupied by Ile (I187), whereas in the other archaeal aquaporins it is occupied either by Ile, Val or Leu [24,26] generating an SF that is narrower than the SF of aquaglyceroporins but wider and less polar than the SF of strict aquaporins. Thus, the SF of AqpM can be viewed as a slightly wider and more-hydrophobic version of the SF from strict aquaporins. Regarding the hydrophobic residues that line the pore below the SF (residues highlighted in red in red), it is interesting to note that among the compared aquaporins these residues correspond to Val, Ile or Leu, except for AqpZ that has two aromatic residues instead in two of these nine positions. These considerations underscore the notion that differences in water permeability between aquaporins are most likely related to the size and polarity of the SF resulting from differences in critically located amino acids. AqpM, with its unique SF, clearly differs from the two other sub-families, exhibiting characteristics that are hybrid between an aquaglyceroporin such as GlpF and a strict aquaporins like AqpZ. Whether this differences permits AqpM to carry out different functions or whether it is just a “molecular fossil” representing an early stage in the evolution of the other aquaporins, as previously suggested [24,26,50], is an issue that remains to be determined. However, the apparent absence of aquaporin genes in a large number of microbial genomes [49], together with the assertion that given the small size and resultant large surface-to-volume ratio of individual microbial cells, lipid membranes are sufficiently permeable to support growth without water channels that enhance water permeability [51,52], have opened the debate about possible alternative roles for microbial aquaporins other than simple water transport. These potential roles include osmotic and turgor sensors [50], involvement in freeze tolerance mechanisms [51] and transport of other neutral solutes such as CO_2_, H_2_S, NH_3 _[51].

## Conclusions

We have performed the first molecular dynamics simulation of the archaeal aquaporin AqpM, the most studied representative of a new group of aquaporins that are not easily classifiable within the current functional sub-families of aquaporins. From our MD simulation we have provided estimations for key biophysical parameters of AqpM including: single channel osmotic permeability constant (***p_f_***), single channel diffusive permeability constant (***p_d_***), channel radius, potential water occupancy of the channel and water orientation inside the pore. The results obtained from the MD simulations of AqpM were compared with those obtained by similar simulation methods for the well characterized microbial representatives of the two main aquaporin sub-families, AqpZ and GlpF, a strict aquaporin and an aquaglyceroporin respectively. Our results extend the evidence that the size and polarity of the residues that comprise the SF of aquaporins control much of the selectivity and water permeability of aquaporins and supports the notion that AqpM belongs to a third class of aquaporins that exhibits properties that are intermediate between those of the other two classes. However, it is clear that more experimental and theoretical evidence is needed to assess whether this third class of aquaporins differs from the other sub-families in other functional aspect besides water permeability rates i.e., if it exhibits permeability to other uncharged solutes, related to the specific life style of the organisms in which they are found or whether it could represent an earlier evolutionary form, a molecular fossil, which existed before the divergence of the other two sub-families.

## Methods

### Modelling and simulation

The crystal structure of the AqpM monomer was obtained from the Protein Data Bank [PDB :2F2B] [26]. Hydrogen atoms were added using the psfgen plug-in from VMD v1.86 [53] (considering pH = 7.5 for protonation states). The tetrameric structure of AqpM was generated with VMD v1.86 [53] using the coordinate transformation matrices provided in the PDB file. The simulated periodic cell was constructed using VMD v1.88 [53] and comprised the AqpM tetramer embedded in a pre-equilibrated 1,2-dipalmitoylphosphatidylcholine (DPPC) membrane patch of dimensions 100x100x58 Å. The energy of the crystallographic waters of the monomer was evaluated with the program DOWSER [54], keeping the waters that were located inside the pore. The system was solvated with a water layer of 20Å above and below the membrane. Ions (Na+, Cl-) were randomly placed to neutralize the system, reaching a final concentration of 50mM. The final dimensions of the periodic cell were 100x100x96Å comprising a total of 85,019 atoms. The full system and the structural features of AqpM can be seen in Figure 1.

The system was minimized and subjected to MD for 0.5 ns with fixed protein. The protein was released keeping Cα atoms constrained with a force constant of 5 kcal/mol/Å^2^. The full system was minimized and a slow relaxation procedure was performed in which the constraint applied to Cα atoms of the protein was decreased at a rate of 0.5 kcal/mol/ps until no constraint was applied. Then 21 ns of NPT-MD simulations were performed with the first nanosecond considered equilibration and the last 20 ns used for analysis. The time evolution of Cα - RMSD with respect to the final conformation of the minimization/relaxation procedure is shown for each AqpM monomer in Figure S1(a) of Additional file 1. The fluctuations of Cα atoms during the production run are presented a Cα – RMSF plot for each AqpM monomer in Figure S1(b) of Additional file 1.

The program NAMD [55] with CHARMM27 parameter set [56-58] was used for the simulation. Periodic boundary conditions were imposed and the particle mesh Ewald method [59,60] was used for electrostatic forces calculation. Constant temperature (310K) and pressure (1 atm) were maintained by using Langevin dynamics [61].

### Analysis methods

#### Single channel water permeability constants

The key quantities that characterize transport properties of a water channel are the single-channel permeability constants: the osmotic permeability constant *p_f_* and the diffusive permeability constant *p_d_* (measured in cm^3^ s^-1^) [28-30,37]. In dilute solutions these constants are related to the fluxes *j_s_* and *j_tr_* due to solute (s) and tracer (tr) concentration differences (ΔC) respectively.(1)(2)

Using equilibrium MD simulations both *p_f_* and *p_d_* can be calculated. From the total number of complete permeation events (i.e the number of water molecules that traverse the channel lumen per unit time) *k_0_*[28,29]*p_d_* can be computed using:(3)

Where *v_w_*=*V_w_/N_A_* is the average volume of a water molecule (2.99x10^-23^ cm^3^).

For the estimation of *p_f_* from equilibrium MD simulations the collective diffusion model for water permeation through microscopic channels proposed by Zhu et al [30] was used. In this model, a collective coordinate can be defined by accumulating at time *t* the individual displacement along the channel axis (*dz_i_*) of all water molecules inside the constriction region (CR) of the channel of average length  relative to *t-δt*. Thus:(4)

By setting *n*=*0* at *t*=*0*, *n*(*t*) can be determined by the integration of *dn*:(5)

At equilibrium *n*(*t*) can be described as a one-dimensional unbiased random walk. By computing the mean square displacement (MSD) of *n*, , the collective diffusion constant can be obtained [30]:(6)

in units of t^-1^, leading to the single-channel osmotic permeability constant *p_f_*[30].(7)

#### Water orientation

To characterize the equilibrium water orientation inside the AqpM channel two order parameters based on Legendre Polynomials where used:(8)

and(9)

where *θ* is the angle between the membrane normal, along the z-axis, and the dipole moment of water. *z* is the position of the oxygen atom of water along the channel axis.

#### Average structure of AqpM

The average structure considered for the structural alignment presented in section **Structure / Sequence Comparisons** (Results and discussion) and in Figure 7 was obtained by averaging the Cartesian coordinates of all the atoms of one monomer of AqpM from the last nanosecond of the simulation at a 2ps resolution (500 frames).

## ^†^AqpM numbering

Residue in one letter code followed by the number in the AqpM sequence. Between parenthesis, the residue in one letter code and number of the equivalent residue of AqpZ and GlpF. Ej: AqpM(AqpZ/GlpF).

## List of abbreviations

CR: Constriction Region, SF: Selectivity Filter, MD: Molecular Dynamics.

## Competing interests

The authors declare that they have no competing interests.

## Authors' contributions

The experimental work was carried out by RAS and JG. DH and TPA conceived the project. The paper was written by RAS with input from TPA and DH. All authors helped interpret the results and read and approved the paper.

## Supplementary Material

Additional file 1 **Cα-RMSD and Cα-RMSF of the simulated AqpM tetramer.** This figure contains two panels: (a) Time evolution of Cα-RMSD for each AqpM monomer obtained from the 20ns production run. The last conformation obtained from the minimization-relaxation procedure (see methods) was used as reference structure for the measurement. (b) Cα-RMSF for each AqpM monomer obtained from the 20ns production run. Each colored solid line represents a monomer. The dashed black line represents the average RMSF. The solid lines below the curves indicate secondary structure elements of AqpM ( blue = TM-helix; orange = loops; green = loops B and E; red = NPA motifs; black = helix B and E). Light blue arrows indicate residues that comprise the selectivity filter (S.F) of AqpM; red arrows indicate hydrophobic residues that line the AqpM pore. Click here for file
