# Supplementary material for: Molecular dynamics study of the archaeal aquaporin AqpM
Source: BMC Genomics. 2011 Dec 22;12(Suppl 4):S8. doi: 10.1186/1471-2164-12-S4-S8 (PMC3287591; doi:10.1186/1471-2164-12-S4-S8)
Supplement: Additional file 1 — Cα-RMSD and Cα-RMSF of the simulated AqpM tetramer. This figure contains two panels: (a) Time evolution of Cα-RMSD for each AqpM monomer obtained from the 20ns production run. The last conformation obtained from the minimization-relaxation procedure (see methods) was used as reference structure for the measurement. (b) Cα-RMSF for each AqpM monomer obtained from the 20ns production run. Each colored solid line represents a monomer. The dashed black line represents the average RMSF. The solid lines below the curves indicate secondary structure elements of AqpM ( blue = TM-helix; orange = loops; green = loops B and E; red = NPA motifs; black = helix B and E). Light blue arrows indicate residues that comprise the selectivity filter (S.F) of AqpM; red arrows indicate hydrophobic residues that line the AqpM pore. [file 1471-2164-12-S4-S8-S1.pdf]

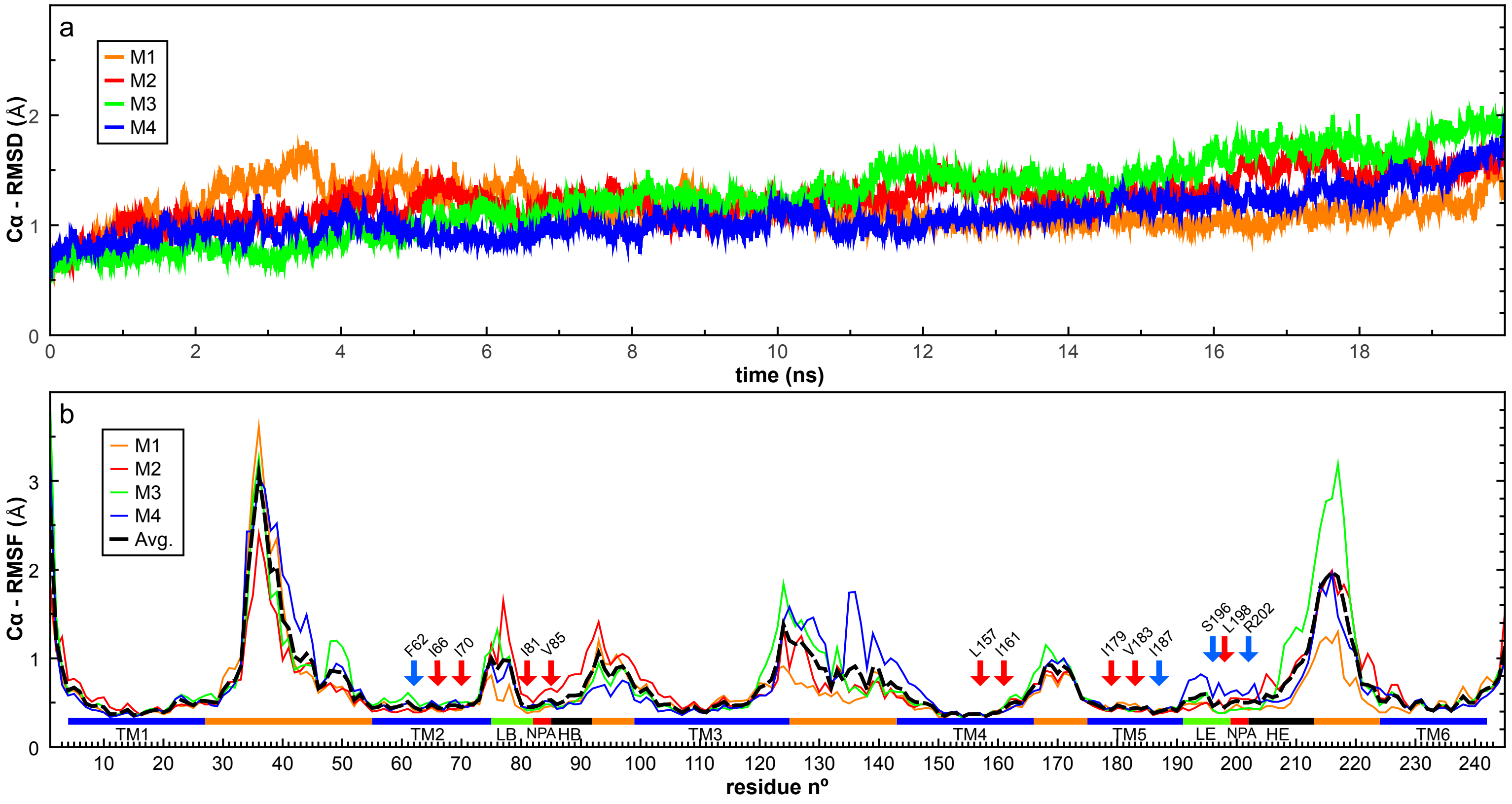


**Fig S1. Cα-RMSD and Cα-RMSF of the simulated AqpM tetramer.**

(a) Time evolution of Cα-RMSD for each AqpM monomer obtained from the 20ns production run. The last conformation obtained from the minimization-relaxation procedure (see methods) was used as reference structure for the measurement. (b) Cα-RMSF for each AqpM monomer obtained from the 20ns production run. Each coloured solid line represents a monomer. The dashed black line represents the average RMSF. The solid lines below the curves indicate secondary structure elements of AqpM ( blue = TM-helix; orange = loops; green = loops B and E; red = NPA motifs; black = helix B and E). Light blue arrows indicate residues that comprise the selectivity filter (S.F) of AqpM; red arrows indicate hydrophobic residues that line the AqpM pore.
